# Supplementary material for: Integrated Analysis of the Transcriptome and Microbial Diversity in the Intestine of Miniature Pig Obesity Model
Source: Microorganisms. 2024 Feb 10;12(2):369. doi: 10.3390/microorganisms12020369 (PMC10891586; doi:10.3390/microorganisms12020369)
Supplement: Supplementary file 1 [file microorganisms-12-00369-s001.zip › microorganisms-2848067-supplementary.pdf]

Integrated Analysis of the Transcriptome and Microbial Diversity in the Intestine of Miniature Pig Obesity Model

Wenjing Qi [1], Siran Zhu [1], Lingli Feng [1], Jinning Liang [2], Xiaoping Guo [2], Feng Cheng [1], Yafen Guo [1], Ganqiu Lan [1,*], Jing Liang [1,*]

[1] College of Animal Science and Technology, Guangxi University, Nanning 530004, China
[2] Laboratory Animal Center, Guangxi Medical University, Nanning 530021, China
* Correspondence: gqlan@gxu.edu.cn (G. L.); liangjing@gxu.edu.cn (J. L.)

Methods

Transcriptome sequencing

Validation using qRT-PCR

Microbial diversity analysis

Figure caption

Figure S1 Unique genera to the jejunum, ileum and colon in ND and HFHCD group.

Table caption

Table S1 Primer sequences used for qRT-PCR

References

Methods

1 Transcriptome sequencing

1.1 Construction and sequencing of mRNA library

Total RNA was extracted from the tissue samples using QIAzol Lysis Reagent (Qiagen, Hilden, Germany). The concentration and purity of the extracted RNA were assessed using Nanodrop 2000 (Thermo Fisher Scientific, Waltham, MA, USA). RNA integrity was evaluated by agarose gel electrophoresis (TaKaRa, Kusatsu, Shiga, Japan), and the RIN value was determined using a 5300 Fragment Analyzer System (Agilent, Santa Clara, California, USA). For library construction, a

minimum of 1 μg total RNA was required, with a concentration ≥ 35 ng/μL, RIN > 6.5, and OD260/280 ratio between 1.8 – 2.2. The TruSeq™ RNA sample prep Kit (Illumina, San Diego, CA, USA) was used for library preparation. The mRNA was sequenced on the Novaseq 6000 platform (Illumina, San Diego, CA, USA).

1.2 Data analysis

After filtering the raw data, clean sequencing data was obtained and aligned with the reference genome (Sscrofa11.1) to acquire the mapped data. The software RSEM[1] was used to analyze the gene expression and transcript levels quantitatively. Subsequently, the DEGs between the samples were identified using the DESeq2 software[2]. A gene with statistically significant differential expression, defined by a P value < 0.05 and $|\log2FC| \geq 1$, was considered a DEG.

1.3 Enrichment Analysis of DEGs

Gene Ontology (GO) annotation and enrichment analysis were performed using Goatools (https://github.com/tanghaibao/GOatools). The statistical significance was calculated employing Fisher's exact test. The Bonferroni correction was applied to adjust the P values, and adjusted P-values ≤ 0.05 were considered significantly enriched for the corresponding GO function. Kyoto Encyclopedia of Genes and Genomes (KEGG) pathway enrichment analysis was conducted using KOBAS[3], following the same principle as the GO functional enrichment analysis. Gene set enrichment analysis (GSEA) was performed using GSEA Version 3.0 (http://software.broadinstitute.org/gsea/index.jsp).

2 Validation using qRT-PCR

The MiniBEST Universal RNA Extraction Kit (TaKaRa, Kusatsu, Shiga, Japan) was used to extract the total RNA. The concentration, purity, and integrity of the RNA were determined as described in section 2.6. Reverse transcription was performed using the PrimeScript™ RT reagent Kit with gDNA Eraser (Perfect Real Time) (TaKaRa, Kusatsu, Shiga, Japan). qRT-PCR was performed using the TB Green® Premix Ex Taq™ II (TaKaRa, Kusatsu, Shiga, Japan) on a CFX96 system (Bio-Rad, Hercules, CA, USA). GAPDH was used as a reference gene, and the primers used are shown in Table S1.

3 Microbial diversity analysis

The total DNA was extracted using the PF Mag-Bind Stool DNA Kit (Omega Bio-Tek, USA). The purity, concentration and integrity of DNA were assessed with NanoDrop2000 (Thermo Fisher Scientific, USA) and 1% agarose gel electrophoresis (Thermo Scientific, USA). PCR amplification of the 16S V3-V4 region was performed using upstream primer 338F: ACTCCTACGGGAGGCAGCAG and downstream primer 806R: GGACTACHVGGGTWTCTAAT (Bio-Rad, USA). After the PCR products were identified, purified, quantified and normalized, a PE library was constructed and sequenced by Illumina. Original sequence quality control was conducted using fastp[4] (https://github.com/OpenGene/fastp, version 0.20.0), and assembly was achieved with FLASH[5] (http://www.cbcb.umd.edu/software/flash, version 1.2.11). The DADA2[6] plugin in Qiime2[7] process was used to reduce the noise of the optimized sequence after quality control and splicing to obtain ASVs (Amplicon Subsequence Variant). Based on the Sliva 16S rRNA gene database (v138), ASVs were classified using the classify-sklearn classifier in Qiime2. The Alpha diversity index was calculated by mothur[8] (http://www.mothur.org/wiki/Calculators， versionv1.30.2), and the inter-group differences of Alpha diversity, community composition and KEGG functional abundance were analyzed via the Wilcoxon rank-sum test. PCoA (Principal Co-ordinates Analysis) based on abund-jaccard distance algorithm was used to test the similarity of microbial community structure among samples, and the PERMANOVA non-parametric test was combined to evaluate significant differences in microbial community structure between sample groups. LEfSe[9] analysis (Linear discriminant analysis Effect Size) (http://huttenhower.sph.harvard.edu/LEfSe) (LDA>2, $P<0.05$) identified bacterial groups with significant differences in abundance from the phylum to genus level across various groups. The data were analyzed on the online platform of Majorbio Cloud Platform (https://cloud.majorbio.com).

Figure caption

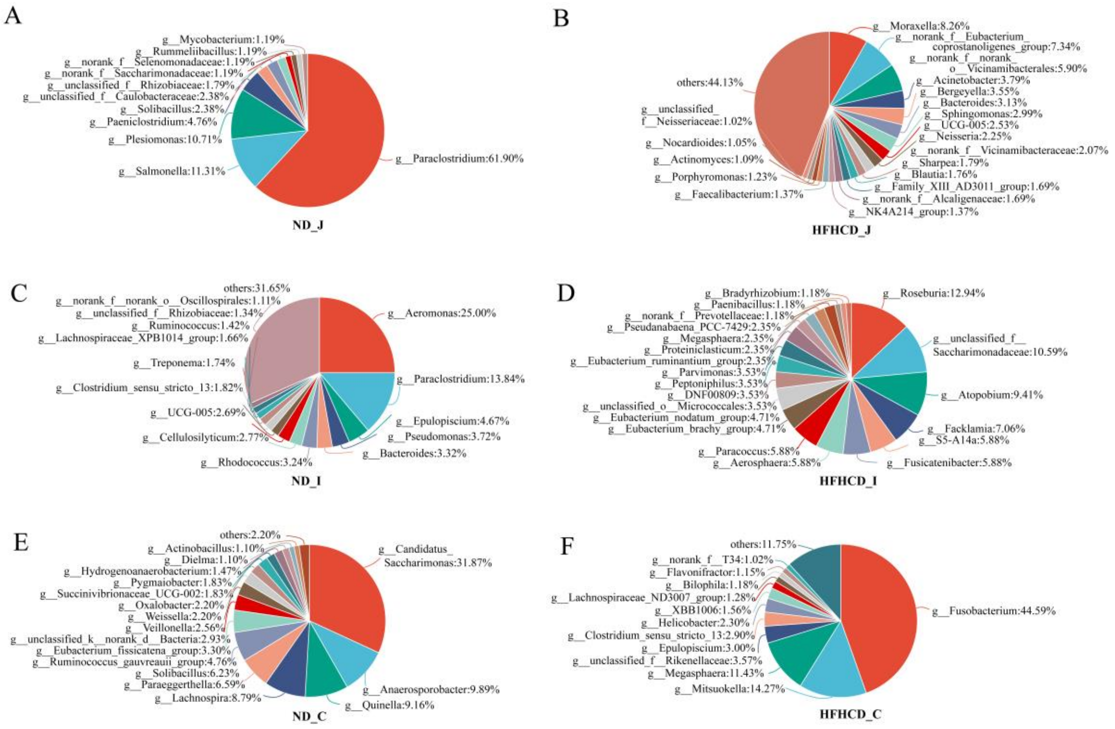

Figure S1 Unique genera to the jejunum, ileum and colon in ND and HFHCD group. (A) Endemic genera to the jejunum in ND group. (B) Endemic genera to the jejunum in HFHCD group. (C) Endemic genera to the ileum in ND group. (D) Endemic genera to the ileum in HFHCD group. (E) Endemic genera to the colon in ND group. (F) Endemic genera to the colon in HFHCD group.

Table caption

Table S1 Primer sequences used for qRT-PCR

Gene Symbol	Gene ID	Primers	Length
COL6A6	ENSSSCG00000037430	TTTTGCTATGATGTGTGC;<br>AGGTGGTGAGATTGAACT	292bp
CYP1A1	ENSSSCG00000001906	ATCCCTATCCTCCGTTACCT;<br>TTCTCGTCCATCCTCTTGTC	182bp
EIF2AK2	ENSSSCG00000008496	GCACCTACATTTACCTCGCC;<br>CATAATCATTCCCATCCCAG	268bp
NMI	ENSSSCG00000022011	AGCACTTATCACCTTTG;<br>CTTCACCTTGACACCCT	91bp
LGALS3BP	ENSSSCG00000036383	ACCTCTTTGCCGTCCTCA;<br>TCCACCAAGCCCTCCACT	201bp

References

1. Li, B.; Dewey, C.N. RSEM: Accurate Transcript Quantification from RNA-Seq Data with or without a Reference Genome. *BMC Bioinformatics* **2011**, *12*, 323, doi:10.1186/1471-2105-12-323.
2. Love, M.I.; Huber, W.; Anders, S. Moderated Estimation of Fold Change and Dispersion for RNA-Seq Data with DESeq2. *Genome Biol* **2014**, *15*, 550, doi:10.1186/s13059-014-0550-8.
3. Xie, C.; Mao, X.; Huang, J.; Ding, Y.; Wu, J.; Dong, S.; Kong, L.; Gao, G.; Li, C.-Y.; Wei, L. KOBAS 2.0: A Web Server for Annotation and Identification of Enriched Pathways and Diseases. *Nucleic Acids Res* **2011**, *39*, W316–W322, doi:10.1093/nar/gkr483.
4. Chen, S.; Zhou, Y.; Chen, Y.; Gu, J. Fastp: An Ultra-Fast All-in-One FASTQ Preprocessor. *Bioinformatics* **2018**, *34*, i884–i890, doi:10.1093/bioinformatics/bty560.
5. Magoč, T.; Salzberg, S.L. FLASH: Fast Length Adjustment of Short Reads to Improve Genome Assemblies. *Bioinformatics* **2011**, *27*, 2957–2963, doi:10.1093/bioinformatics/btr507.
6. Callahan, B.J.; McMurdie, P.J.; Rosen, M.J.; Han, A.W.; Johnson, A.J.A.; Holmes, S.P. DADA2: High-Resolution Sample Inference from Illumina Amplicon Data. *Nat Methods* **2016**, *13*, 581–583, doi:10.1038/nmeth.3869.
7. Bolyen, E.; Rideout, J.R.; Dillon, M.R.; Bokulich, N.A.; Abnet, C.C.; Al-Ghalith, G.A.; Alexander, H.; Alm, E.J.; Arumugam, M.; Asnicar, F.; et al. Reproducible, Interactive, Scalable and Extensible Microbiome Data Science Using QIIME 2. *Nat Biotechnol* **2019**, *37*, 852–857, doi:10.1038/s41587-019-0209-9.
8. Schloss, P.D.; Westcott, S.L.; Ryabin, T.; Hall, J.R.; Hartmann, M.; Hollister, E.B.; Lesniewski, R.A.; Oakley, B.B.; Parks, D.H.; Robinson, C.J.; et al. Introducing Mothur: Open-Source, Platform-Independent, Community-Supported Software for Describing and Comparing Microbial Communities. *Appl Environ Microbiol* **2009**, *75*, 7537–7541, doi:10.1128/AEM.01541-09.
9. Segata, N.; Izard, J.; Waldron, L.; Gevers, D.; Miropolsky, L.; Garrett, W.S.; Huttenhower, C. Metagenomic Biomarker Discovery and Explanation. *Genome Biol* **2011**, *12*, R60, doi:10.1186/gb-2011-12-6-r60.
